# Review on the Applications of Selected Metal-Based Complexes on Infectious Diseases

**DOI:** 10.3390/molecules29020406

**Published:** 2024-01-14

**Authors:** Nondumiso P. Dube, Maxwell Thatyana, Ntebogeng S. Mokgalaka-Fleischmann, Ahmed M. Mansour, Vuyelwa J. Tembu, Amanda-Lee E. Manicum

**Affiliations:** 1 Department of Chemistry, Tshwane University of Technology, 175 Nelson Mandela Drive, Private Bag X680, Pretoria 0001, South Africa; dubendumi@gmail.com (N.P.D.); thatyana.maxwell@gmail.com (M.T.); mokgalakans@tut.ac.za (N.S.M.-F.); tembuvj@tut.ac.za (V.J.T.); 2 Department of Chemistry, United Arab Emirates University, Al-Ain 15551, United Arab Emirates; mansour@sci.cu.edu.eg

**Keywords:** organometallic complexes, tuberculosis, coronavirus, antiparasitic, antibacterial, antiviral, mechanism of action

## Abstract

Fatalities caused by infectious diseases (i.e., diseases caused by parasite, bacteria, and viruses) have become reinstated as a major public health threat globally. Factors such as antimicrobial resistance and viral complications are the key contributors to the death numbers. As a result, new compounds with structural diversity classes are critical for controlling the virulence of pathogens that are multi-drug resistant. Derivatization of bio-active organic molecules with organometallic synthons is a promising strategy for modifying the inherent and enhanced properties of biomolecules. Due to their redox chemistry, bioactivity, and structural diversity, organometallic moieties make excellent candidates for lead structures in drug development. Furthermore, organometallic compounds open an array of potential in therapy that existing organic molecules lack, i.e., their ability to fulfill drug availability and resolve the frequent succumbing of organic molecules to drug resistance. Additionally, metal complexes have the potential towards metal-specific modes of action, preventing bacteria from developing resistance mechanisms. This review’s main contribution is to provide a thorough account of the biological efficacy (in vitro and in vitro) of metal-based complexes against infectious diseases. This resource can also be utilized in conjunction with corresponding journals on metal-based complexes investigated against infectious diseases.

## 1. Introduction

Over the last three decades, notable advancements and attempts have been made to stop the spread of infectious illnesses worldwide. These initiatives are focused on the ongoing research and cutting-edge breakthroughs in drug discovery, particularly in the pharmaceutical industries and academic institutions [1,2]. Fungi, viruses, bacteria, and parasites cause what is known as infectious diseases, and they are responsible for numerous mortalities worldwide. Infectious diseases can be divided into two categories, namely emerging (new diseases) or re-emerging diseases (infections that are not new but display issues of drug resistance upon reappearing) [3,4]. Although some infectious diseases, such as polio, Guinea worm disease, lymphatic filariasis, etc., are now thought to be curable, others, such as Chagas disease, sleeping sickness, HIV, malaria, tuberculosis (TB), and COVID-19, still pose a threat to people around the world [2,3].

Specific debilitating conditions including COVID-19, sleeping disease, and tuberculosis are briefly detailed to emphasize the significance of developing alternative therapeutic medications for managing infectious diseases. First off, the feared new coronavirus (SARS-CoV) outbreak that causes COVID-19 (with potential for severe respiratory symptoms or death) has sparked an unparalleled global health crisis that the World Health Organization estimated would result in 3 million fatalities by the year 2020 [5,6]. There are no pharmacological treatments that have been shown to be effective, despite the world’s challenges with the coronavirus unchecked proliferation and its significant effects on healthcare. The greatest challenge confronting medicinal chemists and researchers today is the urgent need to find novel, innovative medication candidates to combat SARS-CoV-2 [7].

Second, the *Trypanosoma brucei gambiense* subspecies accounted for almost 95% of the 992 cases recorded in 2019 and the sleeping disease is still difficult to cure and detect [8]. The major obstacles to treating this condition are the lack of qualified personnel and the limitations of therapeutic approaches. It can be cured with medication if diagnosed early; however, it is fatal if left untreated. Suramin, pentamidine, melarsoprol, and eflornithine are the primary drugs used in Sub-Saharan Africa to treat sleeping sickness [9]. Unfortunately, the current medications have severe side effects and drug resistance, making new treatments for these terrible infections absolutely necessary [10,11,12]. 

Third, with 1.5 million fatalities in 2018, tuberculosis exceeded HIV/AIDS as the leading infectious disease-related cause of death globally [13,14,15]. The emergence of drug-resistant TB variants has made treatment even more complex and challenging [15,16,17]. Only a few treatments are effective against *Mycobacterium bacilli*, and current medications are essentially ineffective against extensively drug resistant and multidrug-resistant *mycobacterium tuberculosis* strains in immune-compromised patients [17]. In order to shorten the duration of treatment, to combat resistant strains without interfering with antiretroviral drugs, and to combat latent bacilli, new drug development techniques must be created [14,17,18].

As was previously stated, microbial/drug resistance and a lack of medications remain major problems in the treatment of infectious disorders [10,19]. It takes very little time for bacteria to become resistant to classes of conventional antibiotics that only include organic derivatives. Most often, resistance has been found shortly after, or even before, the antibiotic was made commercially available for use by humans [20,21]. An appealing method to address the issue of microbial resistance with traditional organic medication candidates is derivatization with metallic fragments (coordination- or organometallic complexes) [20,21]. However, kinetic, and thermodynamic instability, as well as a dearth of logical and systematic structural changes, make coordination complex derivatization problematic [22].

In contrast, organometallic compounds (metal complexes containing at least one metal–carbon bond with unique, remarkable physicochemical properties) are more stable and offer considerable potential with reasonable modification [22]. Therefore, the development of organometallic drugs is a promising strategy in the search for pharmacological remedies for parasitic, viral, and bacterial diseases. In this contribution, the developments in the biological studies of organometallic compound antiparasitic, antibacterial, and antiviral activities are discussed. Additionally, the reported organometallic compounds that were investigated for their antiparasitic, antibacterial, and antiviral properties, and some mechanisms of action, are referenced in this review. The reported data were systematically collected, read, and analyzed from scientific electronic publishers and databases such as Elsevier, ScienceDirect, Scopus, Scifinder, pub Med, and Google Scholar.

### 1.1. Antiparasitic Agents

**Figure 1:**
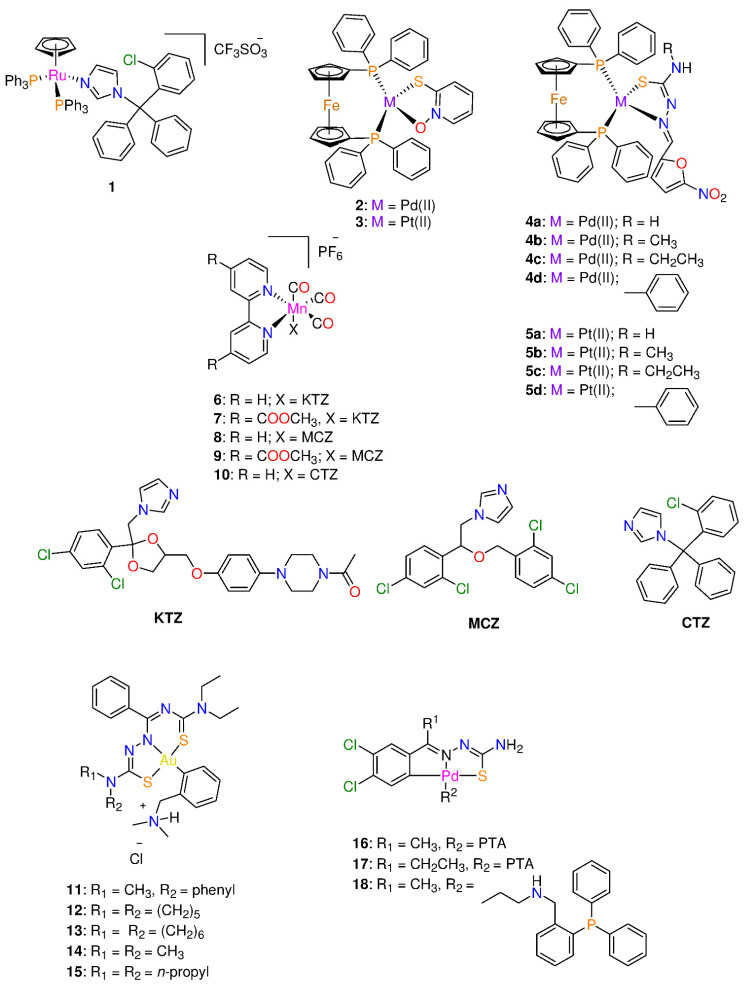
Chemical structure of some organometallic complexes with antiparasitic properties.

Rodríguez Arce et al. [23] evaluated [RuCp(PPh_3_)_2_(CTZ)](CF_3_SO_3_) **1** (Cp: cyclopentadienyl and CTZ: Clotrimazole) (Figure 1) for its antiparasitic properties against some trypanosomatids. CTZ was introduced in the half-sandwich motif as it is commonly used as an antifungal agent for inhibiting fungi from synthesizing sterols. Compound **1** (IC_50_ = 0.25 μM) displayed toxicity against *Trypanosoma cruzi*. The antiparasitic activity of **1** against *T. cruzi* is sixfold increased after CTZ (IC_50_ = 1.8 μM) has been complexed to the ‘RuCp(PPh_3_)’. Additionally, the compound had activity that was around 30 times greater than that of the control substance, **Nifurtimox** (IC_50_ = 8.0 μM) [23,24]. Complex **1** interferes with the biosynthetic process used by *T. cruzi* to turn squalene into squalene oxide. Alternatively, compound **1** showed growth higher inhibitory activity (IC_50_ = 0.6 μM) in *T. bruceibrucei* (strain 427) than CTZ (IC_50_ > 25 μM), generating a dose-dependent antiproliferative impact on the parasites treated for 24 h [23]. Both CTZ and complex **1** were tested for cytotoxicity against a murine macrophage-like cell line (J774) to determine the specificity of their anti-trypanosomal action. Half-sandwich Ru(II) **1** displayed relatively strong selectivity for both parasites (*T. cruzi* (0.25 ± 0.08 µM) and *T. brucei* (0.6 ± 0.1 µM)) over mammalian cells (J774 murine macrophages (1.9 ± 0.1 µM). The selectivity index (SI) data for *T. brucei* show that **1** (SI = 3) is both more potent and more selective than CTZ (SI < 2) [23].

Two complexes of the formula of [M(mpo)(dppf)]PF_6_ (M = Pd(II) **2** and Pt(II) **3**; mpo: pyridine-2-thiolato-1-oxide, and dppf: 1,1′-bis(diphenylphosphino)ferrocene) (Figure 1) were screened for their toxicity against *mycobacterium tuberculosis* (MTB) and *T. cruzi* and VERO epithelial cells (selected as the mammalian cell model) [25]. The ferrocene derivative, dppf, was chosen because it frequently displays low cytotoxicity and gives the molecules in the locations where they are introduced enough lipophilicity to pierce cell membranes [10,25,26]. Both square-planar complexes demonstrated 10–20 times selectivity toxicity against MTB and *T. cruzi* than Nifurtimox, the reference drug (IC_50_ = 6.0 μM) [25,27]. In fact, the cytotoxicity of **2** and **3** was quite high on *T. cruzi* (0.64 ± 0.03 µM and 0.28 ± 0.01 µM) and MTB (2.8 ± 0.7 µM and 1.6 ± 0.3 µM), respectively. Complex **3** is more potent than **2** and the sodium salt of mpo ligand (2.42 ± 0.07 µM) [17,25], revealing the importance of the choice of metal ion in drug design. In addition, the two complexes displayed toxicity in the micromolar range against the epimastigote form of the *T. cruzi*, Dm28c strain. A two- to a five-fold increase of activity was observed for the free Na mpo (IC_50_ = 1.33 ± 0.08 µM) **2** and **3**, respectively [25]. Moreover, the complexes showed about 10–20 times higher activity than Nifurtimox (IC_50_ = 6.0 μM) [25,27]. Hence the inclusion of the ferrocene moiety (dppf ligand) on the metal complexes improved the selectivity towards the parasite [25].

The in vitro cytotoxicity activity on VERO epithelial cells (ATCCCCL81) was assessed. On these cells, the IC_50_ values of **2** and **3** are 24 ± 12 and 5 ± 3 µM, respectively, as well as the selectivity index values being stated in µM units. Molecular docking and experimental studies on *T. cruzi* protein extracts revealed that both **2** and **3** might be acting as effective inhibitors of NADH-fumarate reductase (TcFR) [25].

A concentration-dependent suppression of proliferation was observed when mid-exponential growth phase epimastigotes were incubated with increasing concentrations (0.2–0.7 µM) of **2**. The IC_50_ value (0.30 ± 0.03 µM) [28], determined after 5 days, is nearly twice as low as the IC_50_ reported for the Dm28c strain [25]. This suggests that type VI CL Brener, which has a different genetic ancestry, is more susceptible than the type I Dm28c strain. The SI value of **2** for the CL Brener strain was 83. The cell uptake values were found to be 12% and 16% when the parasites treated with five and ten concentrations of IC_50_ value, respectively [28]. After only 24 h of treatment, compound **2** has a trypanocidal impact, shrinking the parasite’s cell body and triggering necrosis, among other alterations in morphology. When compared to the untreated control infected monolayer, the reduction in intracellular amastigotes was more pronounced after 48 h following incubation.

Further studies on compound **3** (60 ± 3 nM) indicated that the Pt(II) complex has promising anti-trypanosomal activity against CL Brener strain compared to that of Nifurtimox (2.8 ± 0.2 µM) [10,19]. In parasites treated with one and ten concentrations of IC_50_ value, complex **3** uptake reached ~75% and 19%, respectively [10]. This is in contrast with other metal-based antiproliferative compounds such as pyrodach-2 (0.1%), oxaliplatin (1%) or cisplatin (3%) [10,29]. Complex **3** caused necrosis after 24 h of parasite incubation via a substantial reduction of the mitochondrial membrane potential in a way that was dose-dependent [10]. Cell vitality tests on treated parasites showed significant esterase activity. When compound concentration and incubation time were increased, treated epimastigotes showed rounded morphology, flagellum loss, as well as decreased mobility despite having increased metabolic activity. After 48 h of incubation, 3 mM of **3** were sufficient to lower the total amount of amastigotes for each cell by more than 50% [10].

Mosquillo et al. [30] investigated the effect of **2** and **3** on the general transcriptomic process, on *T. cruzi*, since the compounds showed a preference for connecting with DNA among other macromolecules studied. More modified transcripts (2327 of the 10,785 identified transcripts) were produced by **2** treatment than by **3** treatment (201 of the 10,773 identified transcripts), indicating that **2** has a transcriptome-level mechanism of action. Similar numbers of proteins with differential expressions (342 and 411 for **2** and **3,** respectively) were observed. Additionally, transcripts implicated in DNA binding, oxidative defense, transmembrane transport, protein metabolism, and the ergosterol pathways were discovered to be altered by the presence of these complexes [30].

Rodríguez Arce and his co-workers [31], replaced pyridine-2-thiolato-1-oxide with thiosemicarbazone ligands to create two series of Pd(II) and Pt(II) complexes **4** and **5** (Figure 1) that are comparable to the structures of **2** and **3** in their search for novel antileishmanial medications. Compounds **4** and **5** exhibited antiparasitic efficacy against *T. cruzi* trypomastigotes, blood-stream *T. brucei,* and mammalian endothelial cells. Platinum(II) complexes were more potent than palladium analogues. Complexes **5b** and **5c** displayed the largest anti-*T. cruzi* (0.79 and 0.76 μM) and anti-*T. brucei* (IC_50_ = 0.60 μM and 0.52 μM) activities, respectively, among the tested series [31]. In general, the complexes are more potent than Nifurtimox and the free thiosemicarbazone ligands. Coordination of thiosemicarbazone ligands to either Pd(II) or Pt(II) ion reduced toxicity of mammalian cells and increased selectivity towards both parasites. The complexes selectivity indexes for *T. cruzi* and *T. brucei* were in the range 2–66 and 32–83, respectively. Complexes **4** and **5** affect the parasite redox metabolism via interaction with DNA. The most active and selective compound in the new series, **5c** (Figure 1), also demonstrated no in vivo toxicity in zebrafish embryos in the concentration range of 1–100 µM [31].

Five tricarbonyl Mn(I) complexes with the general formula of [Mn(bpy^R,R^)(CO)_3_(X)]PF_6_ (bpy: 2,2’-bipyridine; R = 4-COOCH_3_ and H; X = miconazole (MCZ), ketoconazole (KTZ), and clotrimazole (CTZ)) **6**–**10** (Figure 1) were screened against a variety of parasitic microorganisms and pathogenic bacteria that cause neglected illnesses including sleeping sickness and leishmaniasis [32]. Investigations were made on the antiparasitic activity against *Trypanosoma brucei* and *Leishmania major* and antibacterial activity on eight Gram-positive (*Staphylococcus epidermidis*, *Staphylococcus aureus*, *Enterococcus faecium*, and *E. faekalis*) and Gram-negative (*Yersinia pseudotuberculosa*, *Y. pestis*, *Pseudomonas aeruginosa*, and *Escherichia coli*) bacterial strains was determined. Compounds **6**–**10** exhibited no activity against the Gram-negative bacteria except for **8**, which displayed moderate MIC values of 10–20 μM on *Y. pestis*, *Y. pseudotuberculosa* and *E. coli* and had a stronger activity than the free MCZ ligand (MIC > 40 μM) [32]. However, they demonstrated low to sub-micromolar MIC values on Gram-positive bacteria, particularly on *S. aureus* and *S. epidermidis* with MIC values of 0.625 μM. Complexes **6**–**10** were more active than the parent antifungal azole drugs. The toxicity of **6**–**10** is influenced by the nature of the azole drug coordinated to Mn(I) ion. Among the metal complexes, the activity increases with the type of azole ligand in the order KTZ < MCZ < CTZ [32]. The antileishmanial activity of **6**–**10** was in the 2–5 μM range, but further development was inhibited by comparably high activity on other mammalian cell lines assessed for comparison, resulting in a relatively low selectivity (SI ranges from 1 to 12). Coordination of the azole drugs to Mn(bpy^R,R^)(CO)_3_^I^ moiety resulted in an improvement in the antiparasitic activity against *T. brucei* and *L. major*. In fact, *T. brucei* was more susceptible to **6**–**10** than *L. major*. Complexes **6**–**10** exhibited promising anti-trypanosomal activity, especially compound **6**, which had the greatest activity, with an IC_50_ of 0.7 μM and SI > 10 [32].

Lopes et al. [33] examined the activity of five Au(III) complexes **11**–**15** (Figure 1) in vitro and in vitro. The complexes displayed high aqueous solution stability as well as antiparasitic efficacy toward the *Tulahuen* LacZ strain. In spleen cells, complex **14** showed modest cytotoxicity, and a selectivity index (SI) of about 30 [34]. In addition, the treatment with **11**–**15** (IC_50_ = 0.3 ± 0.06 μM) was more effective against *T. cruzi* trypomastigotes than benznidazole (IC_50_ = 0.84 ± 0.29 mM). The trypanocidal efficacy of benznidazole on *T. cruzi* Y strain intracellular amastigotes, declined with subsequent concentrations (IC_50_ = 1.84 ± 0.5 μM). In contrast, complexes **11**–**15**, at the same concentration, showed a strong trypanocidal activity at all doses tested (IC_50_ = 0.64 ± 0.1 μM). Lopes and co-workers noted that **11**–**15** have the ability to prevent 50% of parasite multiplication at 780 nM, indicating that **11**–**15** are four times more effective than benznidazole, while having no cytotoxic effects on mammalian cells [33]. In phosphate-buffered saline, complexes **11**–**15** turned into the neutral form, which lowered parasitemia and tissue parasitism while preventing tissue damage to the heart and liver at 2.8 mg/kg/day. Upon the neutralization of **11**–**15**, during the acute phase of treatment, 100% of the mice received the gold complex. After 150 days of infection, the parasite load in the acutely infected animals who survived was comparable to those animals treated with the standard dose of benznidazole without displaying the latter’s hepatotoxicity [33]. Additionally, it was discovered that interferon-gamma (IFN-γ) levels might be modulated to target a favorable outcome of the disease [33]. This is the first in vivo experimental model of gold organometallic research on Chagas disease that shows promise.

Adams et al. [35] synthesized cyclopalladated thiosemicarbazone complexes by using phosphorus ligands including PTA and aminophosphines. The aforementioned complexes were developed by breaking the bridging Pd–S bonds of tetranuclear complexes that were previously described [36]. The antiplasmodial properties of the cyclopalladated complexes (**16**–**18**, Figure 1, Table 1) were tested against two strains of *Plasmodium Falciparum*, Dd2 (chloroquine- resistant), and NF54 (sensitive to chloroquine), with inhibitory results at low micromolar concentrations [35,37]. Complexes **16**–**18**, exhibited comparable inhibitory action in both strains, with IC_50_ values between 1.59 and 2.69 μM [35,37]. Since there is promising biological evidence for mononuclear complexes, altering these complexes may increase their activity. 

### 1.2. Antibacterial Agents

**Figure 2:**
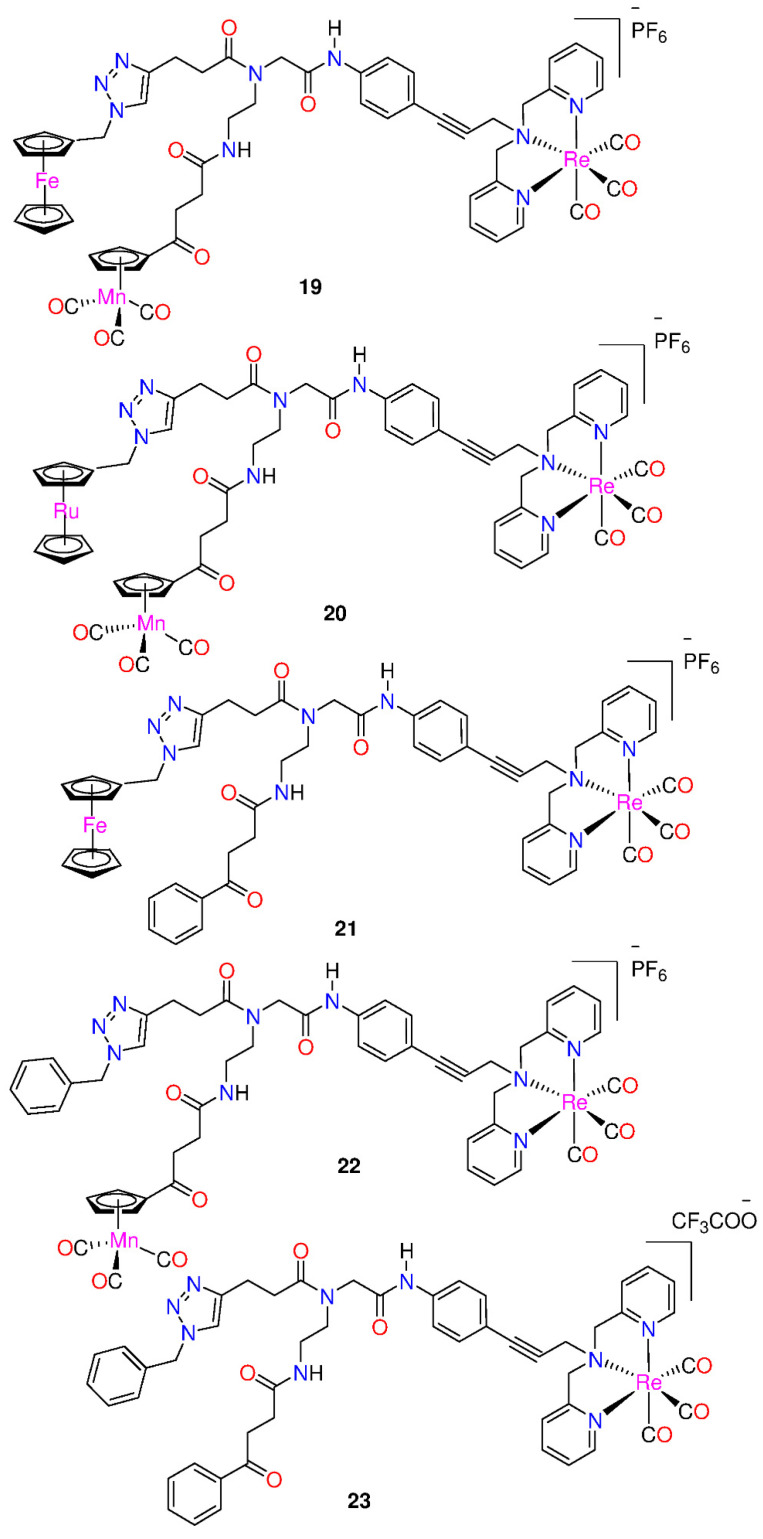
Hetero-tri-organometallic compounds *FcPNA* and *RcPNA* derivatives.

Wenzel et al. [38] reported the powerful antibacterial properties of the hetero-tri-organometallic complex **19** (see Figure 2, Table 2). At MIC of 2 µg mL^−1^ (1.4 µM, Table 2), it prevents the growth of Gram-positive bacteria, including *Staphylococcus aureus* ATCC 43300 (MRSA) [38]. Complex **19** has 90 times the activity against MRSA than the commercial antibacterial drug amoxicillin (MIC = 48 µg mL^−1^, 131 µM) and is equivalent to Norfloxacin (MIC = 0.5 µg mL^−1^, 1.6 µM). A ruthenocene derivative, **20**, was slightly less active than **19,** with MICs in the range of 4 to 32 µg mL^−1^. Both complexes have a high interaction with the bacterial membrane, interfering with membrane-related functions like respiration and cell wall biosynthesis, according to studies on their mode of action [38]. In addition, **19** causes oxidative stress in bacteria, but **20** does not. The pharmacological evaluation of the compounds was constrained by the solubility of both complexes in an aqueous solution, which was approximately 25 µg mL^−1^ [20]. According to Wenzel et al. [38], ferrocene-induced oxidative stress tends to increase antibacterial efficacy but is not the only factor affecting antibiotic activity. 

Derivatives **21** and **22**, demonstrated powerful antibacterial action, with MIC values in the range of 2–4 µg mL^−1^ [20]. The mono-metallic derivative **23**, which had the organometallic [Re(dpa)(CO)_3_] as the only residue while phenyl groups replaced both CpMn(CO)_3_ and ferrocenyl, was discovered to preserve total antibacterial action. It inhibits MRSA development, at a concentration 2 µg mL^−1^. The activity is equivalent to that of the parent compound **19** as well as the bi-metallic derivatives **21** and **22**. According to these findings, neither the ferrocene nor the CpMn(CO)_3_ moiety is essential, but the presence of the Re[(dpa)(CO)_3_] moiety is crucial for antibacterial activity [20]. None of the active compounds **19−23**, had any activity against Gram-negative *Acinetobacter baumannii*, *Escherichia coli,* or *Pseudomonas aeruginosa.* This may be due to the existence of the outer membrane of Gram-negative bacteria, which prevents many compounds from reaching the cytoplasmic membrane, where compound **19** exerts its activity. However, **21−23**, demonstrated considerably more efficient membrane permeabilization ability than **19** [20]. Moreover, all the derivatives displayed higher solubility in culture media than the trimetallic **19**, with **23** having the highest solubility.

**Figure 3:**
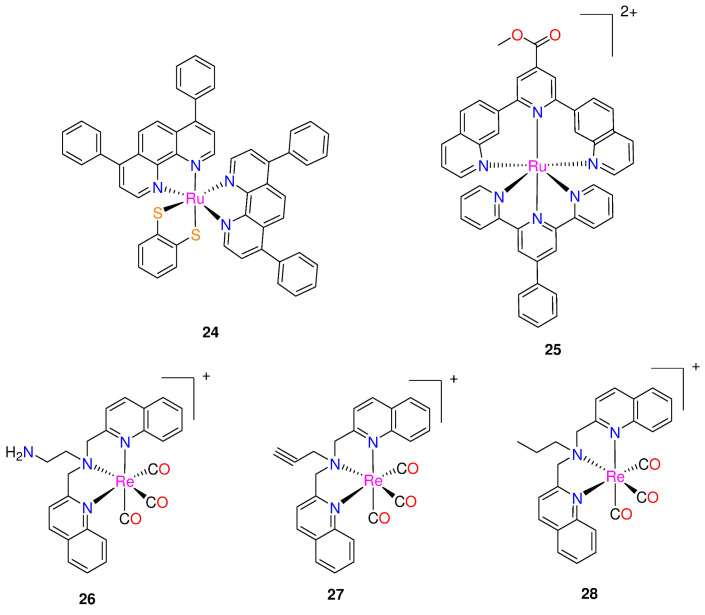
Chemical structures of ruthenium(II) polypyridyl complexes and rhenium(I) bisquinoline complexes.

Frei and co-workers [39] reported two Ru(II) polypyridyl complexes, [Ru(DIP)_2_(bdt)] (**24**) and [Ru(dqpCO_2_Me)(ptpy)]^2+^ (**25**), where DIP = 4,7-diphenyl-1,10-phenanthroline; bdt = 1,2-benzenedithiolate; dqpCO_2_Me = 4-methylcarboxy-2,6-di(quinolin-8-yl)pyridine); and ptpy = 4′-phenyl-2,2′:6′,2″-terpyridine (Figure 3). They were tested for potential antimicrobial photodynamic therapy (aPDT) applications against Gram-positive *Staphylococcus aureus* and Gram-negative *Escherichia coli* bacterial strains. At a light dose of 8 J/cm^2^ (420 nm), both complexes demonstrated significant aPDT activity against *S. aureus*, resulting in a >6 log_10_ CFU reduction at 50 μM concentration. However, at the same light dose and concentration, only complex **25** was able to affect the viability of *E. coli* by >4 log_10_ CFU reduction (99.99% reduced viability) [39]. 

Three rhenium bisquinoline complexes (**26**–**28**, Figure 3), were developed by Frei et al. [40] for aPDT. The bisquinoline scaffold with a tertiary amine was chosen as the tridentate ligand with either alkyne, amine, or alkyl groups. The antibacterial efficacy of the complexes was tested against Gram-positive (*S. aureus* ATCC 25923) and Gram-negative (*E. coli* ATCC 25922) strains, as well as methicillin-resistant *S. aureus* (MRSA) and colistin resistant *E. coli*. These tests were conducted under UV-light (365 nm, 3 J cm^−2^) and in the dark. All the three complexes demonstrated activity against *S. aureus*, with compounds **26** and **27** showing nanomolar to micromolar MIC values, indicating their ability to inhibit the growth of *S. aureus* with and without light irradiation. The MIC were 4- to 16-fold lower when exposed to light, demonstrating that all three compounds had increased activity when exposed to light [40]. No activity (up to 64 µg  mL^−1^) was observed for *E. coli*, in the absence of light. However, upon light irradiation, compound **26** showed MIC values as low as 5.8 μM (4 μg mL^−1^), with modest activity also seen for **27** and **28**. When exposed to light, the complexes demonstrated light-induced antibiotic activities against drug-resistant *S. aureus* and *E. coli* strains, with MIC values ranging between 0.25–8 µg/mL [40]. The researchers examined cytotoxicity of the rhenium complexes against human cells and haemolytic properties. Complex **26** demonstrated the most promising overall activity, exhibiting mild toxicity against human embryonic kidney (HEK293) cells with a CC_50_ of 59.9 ± 9.2 μM, and no hemolysis up to 300 μM [40]. Interestingly, light exposure was found to enhance the cytotoxicity of these compounds. Following 1 h of irradiation with a UV lamp at 365 nm, 3 J cm^−2^, the CC_50_ for **26** decreased to 19.1 ± 5.7 μM. This suggests that complex **26** is generally more toxic against bacteria (13–26 times more effective against *S. aureus* and 1.5–3 times more effective against *E. coli*) than against human cells, even with light-irradiation [40]. Furthermore, it appears that **26**–**28** may possess two modes of activity, one of which is associated with the generation of ROS possibly leading to destabilization of Fe–S clusters and an increase in aminoglycoside uptake [40].

**Figure 4:**
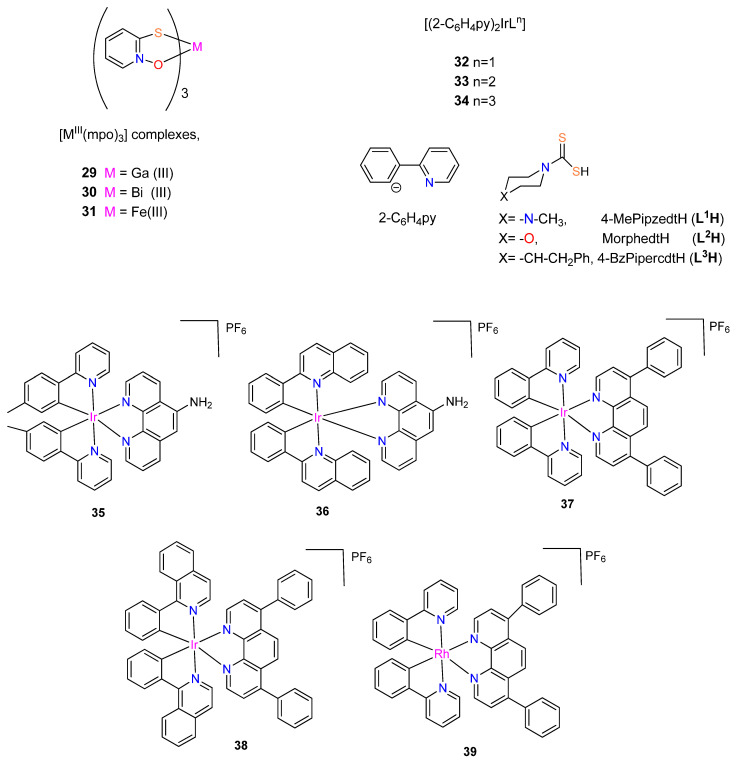
Chemical structure of [M^III^(mpo)_3_] complexes, with M = Ga, Fe or Bi, the corresponding ligands and rhodium(III) or iridium(III) complexes.

Machado et al. synthesized and characterized two [M^III^(mpo)_3_] complexes, where mpo = pyridine-2-thiolato 1-oxide and M = Ga (**29**) or Bi (**30**) (Figure 4) [17]. Both complexes, along with the analogous [Fe^III^(mpo)_3_] compound (**31**), were biologically tested in the standard strain H37Rv ATCC 27,294 (pan-susceptible), as well as in five clinical isolates that are resistant to the conventional first line antituberculosis medications, isoniazid and rifampicin. The compounds demonstrated excellent effectiveness against the resistant and sensitive strains of *M. tuberculosis* [17]. The four complexes tested in vitro had MICvalues that ranged from 1.06 µM to 3.29 µM (Table 3). The complexes exhibited equivalent or more significant anti-bacillus activity than drugs currently utilized in treatment. The World Health Organization’s recommended medications used in the first phase of basic treatment, include rifampicin (MIC value around 0.49 µM), isoniazid (MIC = 0.18 µM), pyrazinamide (MIC value varied in the range of 48.74–406.14 µM at pH 5.5), and ethambutol (MIC = 2.45 µM) [17,25].

In the in vitro assay, complex **29** was the most potent among the compounds tested, eradicating 90% of the bacteria at 1.06 µM concentration [17]. This value is comparable to Phase II clinical development medications, such as SQ109. With an MIC value of 3.29 µM, complex **30** showed less activity but nevertheless qualifies as a strong candidate for a novel anti-TB drug as it exhibits greater activity than pyrazinamide (48.74 µM) and kanamycin (3.43 µM) [17]. The cytotoxicity of the two most promising metal compounds, **29** and **31** on VERO epithelial cells (ATCC CCL81), was in vitro assayed to gain further insight into their potential. The IC_50_ values for these cells as well as the selectivity indexes were evaluated: IC_50_ Vero cells **29** (10.04 µM), **31** (4.61 µM); SI **29** (9.37), **31** (3.03) [17]. Complex **29** was identified as the compound with the most promise for further drug development.

Three mononuclear cyclometalated iridium(III) complexes bearing dithiocarbamate derivatives were synthesized by Mukherjee et al. [42] (see Figure 4). [Ir(2-C_6_H_4_py)_2_(L)] (where 2-C_6_H_4_py = 2-phenylpyridine; and L^1^H = 4-MePipzcdtH, L^2^H = MorphcdtH, and L^3^H = 4-BzPipercdtH for **32**–**34**, respectively). These complexes were synthesized from [Ir(2-C_6_H_4_py)_2_Cl]_2_·1/4CH_2_Cl_2_ by substituting the two bridging chlorides with a single dithiocarbamate ligand [42]. The investigation of the complexes’ antibacterial properties against *Escherichia coli*, *Streptococcus pneumoniae*, *Vibrio cholera*, and *Bacillus cereus* was performed using agar disk diffusion. These cyclometalated iridium(III) complexes interact with CT-DNA to show that they are effective intercalators to CT-DNA with a sufficient number of coordination sites, and the antibacterial studies indicate that all the iridium(III) complexes show stronger activities against four harmful bacteria (*V. cholerae*, *E. coli*, *S. pneumoniae*, and *B. cereus*) than the free dithiocarbamic acids (LH) [42]. Among these three complexes, **33** possesses the strongest antibacterial activity [42]. The complexes would become more lipophilic and easier to penetrate bacterial cell membranes if the *π*-electrons over the chelate ring were to delocalize, thereby inhibiting bacteria from growing [42]. The dithiocarbamic acids (HL) and the iridium(III) complexes antibacterial activity are given in Table 4.

Lu et al. [43] synthesized organometallic complexes with the general structure [M(CN)_2_(*N*,*N*’)]^+^ (M = Ir or Rh) for iridium(III) complexes **35–38** and rhodium(III) complex **39** that are kinetically inert. Complexes **35** and **36** include a 5-amino-1,10-phenanthroline *N*,*N*’-ligand, while complexes **37–39** have the bathophenanthroline*N*,*N*’-ligand [43]. Furthermore, complexes **37** and **39** possess 2-phenylpyridine ligands regarding the CN-ligand, whereas complex **35** has the related 2-(*p*-tolyl)pyridine ligand. In addition, the larger 2-phenylquinoline and 1-isoquinoline CN-ligands, are carried by **36** and **38**, respectively, as shown in Figure 4 [43]. Complexes **36** and **39** were synthesized for the first time by Lu et al. [43], whereas complexes **35** [44], **37** [45], and **38** [46] were previously described.

The complexes **35**–**39** were tested for anti-bacterial activity against four different strains of bacteria (*Enterococcus faecalis*, *Escherichia coli*, *Staphylococcus aureus*, and *Klebsiella pneumoniae*). With an average inhibition zone of 15 mm, the racemic complex **35** (*rac*-**35**) displayed selective anti-bacterial efficacy against *S. aureus* [43]. The iridium(III) complex **35** suppressed *S. aureus* growth with MIC and MBC values of 3.60 and 7.19 μM, respectively, demonstrating its strong bactericidal action. This cyclometallated iridium(III) complex, which is used as a direct and specific *S. aureus* inhibitor, is the first instance of a substitutionally-inert Group 9 organometallic complex [43]. Complexes **36**–**39** showed limited antibacterial activity [43]. 

**Figure 5:**
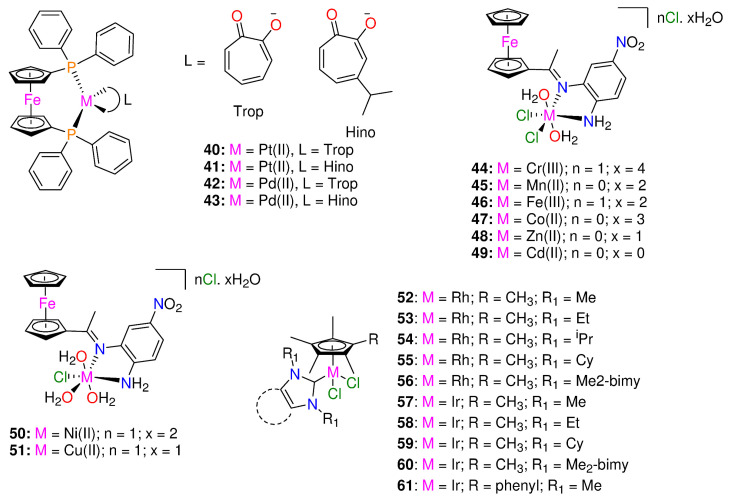
Chemical structures of ferrocenyl derivatives, organometallic Schiff base metal complexes and Rh^III^ and Ir^III^ N-heterocyclic carbene piano-stool complexes.

To design potential metal-based therapies for the management of diseases brought on by tuberculosis and trypanosomatids, Rivas et al. [47] synthesized and fully characterized four new ferrocenyl derivatives, 1,1′-bis(dipheny1phosphino) ferrocene hexafluorophosphate compounds [M(Hino)(dppf)](PF_6_) and [M(Trop)(dppf)](PF_6_), where M = Pt(II) or Pd(II) (Figure 5). The four complexes and ligands were assessed for their biological activities against *T. brucei*, *M.tuberculosis* (MTB) and *L. infantum*, and on human lung cells and murine macrophages. The complexes have significantly stronger anti-*T. brucei* activity than that of the free ligands with IC_50_ values < 5 μM. Additionally, DNA interactions and impacts on *T. brucei* redox metabolism were investigated and fluorescence measurements suggested that DNA is a likely target of the new compounds. 

With IC_50_ values < 5 μM, the heterobimetallic ferrocenyl complexes significantly increased the anti-*T. brucei* activity for the free ligands (>28- and >46-fold for Trop (**40**, **42**) and 6- and 22-fold for Hino (**41**, **43**) coordinated to Pt-dppf and Pd-dppf, respectively) [47]. The complexes were more effective than, Nifurtimox. The novel ferrocenyl analogues were more parasite-selective than the free ligands. Pd(II) complexes (**42**, **43**) (IC_50_ values ranging from 1.2 to 1.3 μM) were slightly more cytotoxic (1.6- to 3.8-fold) against parasites than Pt(II) complexes (**40**, **41**) (IC_50_ values ranging from 2.1 to 4.5 μM), and all Pd(II) complexes were found to be approximately 10-fold more powerful than Nifurtimox [47]; **Nifurtimox** (IC_50_ values: *T. brucei*= 15 ± 3 μM; murine macrophage = 150 ± 5 μM) [48].

The free ligands, HTrop (IC_50_ 67 μM) and dppf (IC_50_ 45 μM) displayed comparable toxicity against macrophages, while HHino is not toxic even at 100 μM. Independently of the ligand, complexation with [Pd-dppf] enhanced the cytotoxicity of the analogues for the free ligands in the following sequence: complex **43** (>30-fold cytotoxic) > **42**(>6.7-fold cytotoxic) [47]. On J774 murine macrophages, the Pt(II) compounds (**40**, **41**) were less toxic than those containing Pd(II) (**42**, **43**), with selectivity index values of up to 23 [47]. All of the synthesized derivatives had anti-leishmanial activity at least two orders of magnitude less potent than that of the reference medication, amphotericin B [47].

The ferrocenyl analogues demonstrated more activity on sensitive MTB compared to the free ligands (MIC_90_ = 9.88–14.73 μM) and displayed minimal pathogen selectivity [47]. The calculated SI values against the human lung fibroblasts MRC-5 were ≤1. When compared to the sensitive strain (H37Rv), The complexes efficacy against the clinical strains was lower [47]. The HHino ligand, as well as the Pt-dppf and Pd-dppf derivatives (MIC_90_ = 13.1–35.4 µM) were active against the susceptible strain (H37Rv); however they were ineffective against the resistant variants (MIC_90_ > 25 µM). The most effective analogues against the triple resistant TB strain CF100 were HTrop (MIC_90_ = 18.30 μM) and **42** (MIC_90_ = 9.05 μM), proving their anti-TB potential. The antiparasitic activity cannot be attributed to the complexes interfering with the pathogen thiol-redox homeostasis, according to the mechanism of action [47]. 

A novel bidentate organometallic Schiff base ligand (L), (2-(1-((2-amino-5-nitrophenyl)imino)ethyl)cyclopenta-2,4-dien-1-yl)(cyclopenta-2,4-dien-1-yl)iron and its Cr(III) (**44**), Mn(II) (**45**), Fe(III) (**46**), Co(II) (**47**), Zn(II) (**48**), Cd(II) (**49**), Ni(II) (**50**), and Cu(II) (**51**) complexes (Figure 5), were synthesized by Mahmoud et al. [49]. These were tested for antimicrobial activity against several fungal and bacterial species by utilizing the disc diffusion technique [49]. Complex **49** demonstrated higher activity with an inhibition ratio of 74% against breast cancer and a lower side effect on normal cells than the others, while complex **47**, **48,** and **49** had higher activities against all various bacterial species than the other complexes [49]. The bacterial organisms used in in vitro antibacterial and antifungal activities were Gram-positive bacteria (*Streptococcus mutans*, *Staphylococcus aureus*), Gram-negative bacteria (*Klebsiella pneumonia*, *Pseudomonas aeruginosa*, *Escherichia coli*), and fungal species including (*Candida albicans*). In comparison, complexes **47**–**49**, demonstrated greater activity against all types of bacterial species (inhibition zone diameter = 10.7–40.7 mm/mg) [49]. Complex **48** was the most active compound against *streptococcus mutans* species (40.7 ± 0.6 mm/mg), surpassing the Schiff base ligand (24.0 ± 1.0 mm/mg) and ampicillin standard. No activity was shown by **44**, **46,** and **50** against any species [49]. *Candida albicans* was biologically inhibited by the Schiff base ligand with 13.30 mm/mg. Compared to the standard antifungal medication (Ketoconazole) and the parent ligand, complex **49** was a more potent fungicide. Complex **49** demonstrated fungicidal activity against the same species at 14.30 mm/mg. The other complexes had no effect on *Candida albicans* [49].

To enhance the rapidly expanding interest in metal NHC complexes’ biological characteristics, Bernier et al. [50] synthesized a series of Ir^III^ and Rh^III^ piano-stool complexes of the form [(η^5^-Cp^*R^)M(NHC)Cl_2_] (M = Rh, Ir) (see Figure 5). These types of piano-stool complexes constitute appealing antimicrobial candidates because of their strong facile modularity and water solubility, allowing effortless modification to adapt the complexes toward particular biological uses and targets [50,51]. The complexes’ antibacterial properties were investigated against *Mycobacterium smegmatis*. The majority of the complexes had moderate antimicrobial activity (≤15 μM) to high (≤1 μM) against Gram-positive *Mycobacterium smegmatis* [50]. Rhodium complexes were discovered to have higher activity than iridium complexes, and by using additional hydrophobic Cp*R and NHC ligands, the activity was increased [50]. 

There are a few exceptions to this trend, with the iridium Cp* complex exhibiting higher activity with Me_2_-bimy (**60**) (MIC = 0.92 μM) and the iridium Cp*phenyl complex with IMe (**61**) (MIC = 0.45 μM). Additionally, the activity of the rhodium complexes having more hydrophobic NHC ligands (**53**–**56**,) was significantly increased and it ranged from 0.46 μM to 1.1 μM, compared to that of IMe (**52**), which was 4.9 μM [50]. The iridium Cp* complexes followed the same pattern, with the exception of IEt variant (**58**) (MIC = 31 μM), showing significantly less activity against *M. smegmatis* than **57** (MIC = 8.1 μM), **59** (MIC = 1.6 μM), and **60** (MIC = 0.92 μM) [50]. Furthermore, the introduction of a phenyl-substituted Cp*^R^ into iridium Cp*^R^ complexes containing the IMe NHC ligand improved the performance of the complexes, as **61** (0.45 μM) was discovered to be significantly more powerful than **57** (8.1 μM) and having the lowest MIC of all the tested complexes [50]. The cytotoxicity tests revealed that 55 is only toxic to Calu-3 cell lines and mammalian Vero E6 at 200-fold higher (92 μM) concentrations than the reported activity against *M. smegmatis* 0.25 μg/mL (0.46 μM). These results emphasize the potential for noble-metal piano-stool antimicrobial compounds to be developed and tailored using N-heterocyclic carbene and Cp*^R^ moieties.

### 1.3. Anti-Viral Agents

Five gold organometallics were evaluated as inhibitors of two important therapeutic targets of severe acute respiratory syndrome coronaviruses (SARS-CoV), in a study by Gil-Moles et al. [7]. The effects of the chosen experimental gold metallodrugs were investigated on two appropriate coronavirus targets (spike protein, papain-like protease, PLpro). Complexes **62** [52], **64** [53], and **66** [54] were chosen from prior studies on organometallic gold metallodrugs, whereas the initial reports of **63** and **65** were made by Gil-Moles et al. [7]. The SARS-CoV-2 spike protein interaction with the angiotensin-converting enzyme 2 (ACE2) host receptor was inhibited by the gold metallodrugs, potentially interfering with the viral entry process. The gold complexes **62** to **66** showed good IC_50_ values in the 16–25 µm range, making them marginally more effective than Chloroquine, the reference drug (IC_50_ value: 31.9 µm) [7].

SARS-CoV-1 and SARS-CoV-2 papain-like protease (PLpro)—a critical enzyme in viral replication—were effectively inhibited by the gold metallodrugs. The potency of the reference inhibitor Disulfiram was matched by the IC_50_ values of complexes **62**, **63,** and **66** against PLpro from SARS-CoV-1, in the range 5–7 µm. Complexes **64** and **65** were less active, having IC_50_ values of 14 µm [7]. The gold compounds **62**, **63,** and **66** as well as the reference inhibitor Disulfiram, showed potent inhibitory effect against SARS-CoV-2 PLpro, with IC_50_ values that were near to 1.0 µm. Notably, **64** and **65** had IC_50_ values above 50 µm and were inactive against SARS-CoV-2 PLpro. Complexes **64** and **65** with their moderate activity against SARS-CoV-1 PLpro (14.2 ± 0.3 µm and 14.1 ± 2.1 µm, respectively), behaved differently from the other complexes, which were more effective against SARS-CoV-2 PLpro than SARS-CoV-1 enzyme [7]. With an IC_50_ > 100 µm, benzimidazole was utilized as a negative reference.

Notably, the ability of the inhibitors to remove zinc ions from the enzyme’s labile zinc center correlated with the activity of the complexes against both PLpro enzymes [7]. In SARS-CoV-1 PLpro trials, the most potent inhibitors Disulfiram, **62**, **63**, and **66** were effective zinc ejectors. The zinc-removing action of the moderate SARS-CoV-1 PLpro inhibitors **64** and **65** were strongly time-dependent. Except for **64** and **65**, all compounds were efficient zinc ejectors in the tests using SARS-CoV-2 PLpro [7]. Therefore, gold complexes have the potential to target two important pathways in the coronavirus life cycle [7]. The most potent activity was shown against SARS-CoV-2 PLpro with **62**, **63,** and **66** [7]. Thus, the compounds are among the first highly effective inhibitors of the target enzyme. 

**Figure 6:**
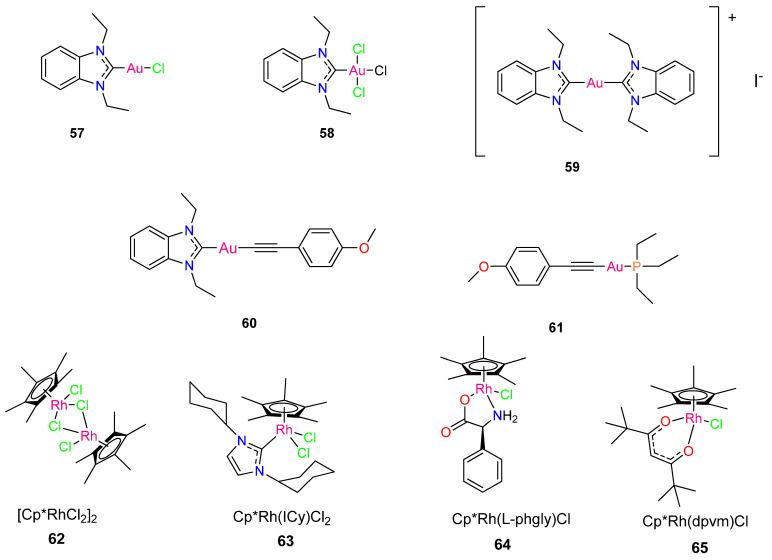
Chemical structure of gold metallodrugs and pentamethylcyclopentadienyl (Cp*) rhodium piano stool complexes.

To develop effective antiviral therapies to tackle the devastating SARS-CoV-2, Chuong et al. [55] evaluated noble metal organometallic compounds for the virucidal activity against the viral pathogen. The complexes include four rhodium complexes indicated as complexes **67**–**70** and are shown in Figure 6. Complexes **67**, **69**, and **70** include the ligands 1,3-dicyclohexylimidazol-2-ylidene (**68**), phenylglycinato (**69**), and dipivaloylmethanato (**70**). The synthesis of each complex as well as its antimicrobial properties were reported previously [50,56,57,58]. Complex **68** and **70** were found to exhibit direct virucidal action against SARS-CoV-2. In addition, subsequent in vitro tests revealed that complex **70** is the most stable and effective complex, demonstrating that both complexes **68** and **70** exhibit minimal toxicity in Vero E6 Calu-3 cells [55].

The activity of four complexes (Figure 6) was tested to inactivate SARS-CoV-2 directly. Chuong et al. [55] chose these specific complexes because they displayed potent antimycobacterial activity against *M. smegmatis* [50,56,57,58]. Two complexes, Cp*Rh(1,3-dicyclohexylimidazol-2-ylidene)Cl_2_ (**68**) and Cp*Rh(dipivaloylmethanato)Cl (**70**), demonstrated direct virucidal efficacy against SARS-CoV-2 [55]. Complexes **68** and **70**, which exhibited the lowest MICs against *M. smegmatis* (0.46 µM and 2.19 µM, respectively), displayed the strongest virucidal activity against SARS-CoV-2 and were considerably more virucidal than complex **67** and **69** at all concentrations analyzed (p < 0.0001). Complex **68** decreased plaque formation by about 84% at 10 µg/mL compared to complex **70** which significantly reduced plaque formation by 98% (p = 0.0021). Complexes **68** and **70** reduced SARS-CoV-2 by >99% at concentrations of 25 and 50 µg/mL [55].

Extended incubation periods of up to 3 h resulted in higher virucidal activity. Complexes **67** and **69** were less effective in suppressing SARSCoV-2 plaque formation, and efficacy did not improve as concentration was increased [55]. Following in vitro studies indicates that complex **70** is the most stable and effective complex, with both **68** and **70** demonstrating minimal toxicity in Vero E6 and Calu-3 cells [55]. The findings emphasize the potential antiviral activity of organometallic complexes and encourage further research into their efficacy.

## 2. Conclusions

The potential for organometallic compounds to cure infectious disorders was proven by the discovery of salvarsan in Ehrlich’s laboratory in the early 1900s, an organo arsenic antimicrobial agent [59]. Even so, only a few organometallic compounds have been used to fight these diseases. To prevent the causative parasites from developing resistance genes and to combat the significant adverse effects of current therapies for these diseases, new therapeutic agents are required. The results presented in this review reinforce the potential and significance of focusing on metal complexes for drug discovery in the battle against infectious diseases that are still inefficiently treated. The organometallic compounds demonstrated suitable biological activities against the different pathogens they were tested on and the mechanisms of action were uncovered.

Recent research has highlighted the significant impact of metal coordination on drug design, showing a marked improvement in the biological activity and expanded medicinal applications of established drugs. Notably, organometallic compounds like Pt-dppf-mpo and gold(III)–thiosemicarbazone have emerged as promising agents with anti-parasitic and anti-chagasic properties, warranting further investigation into their animal toxicity and biodistribution. Moreover, pyridine-2-thiol 1-oxide derivatives have shown promise in treating resistant tuberculosis, indicating potential for the development of new bioactive metal complexes. The encouraging biological data for mononuclear complexes suggest that modifications could enhance their activity. Additionally, Group 9 transition metal complexes are under exploration as therapeutic agents due to their unique geometry and properties, with the [Re(CO)_3_]^+^ core presenting a promising structure for antibacterial compound development. The focus should be on developing easily synthesizable derivatives with improved solubility and biological potency while maintaining selectivity over mammalian cells. These recent findings underscore the exciting potential of organometallic compounds in advancing novel therapeutic applications.

Further physiochemical studies of these compounds and a broader knowledge of the mechanism of action of organometallic drugs need to be exploited. One of the shortcomings in these studies is the absence of in vivo data, which are required to determine whether a compound has any genuine pharmacological potential. Limited in vivo studies on these compounds can be attributed to their high reactivity and heavy metal content, which raises concerns about potential toxicity to living organisms, including humans. Moreover, the complex nature of organometallic compounds and their mechanisms of action may not be fully understood, making it difficult to predict their efficacy and potential side effects. Nevertheless, the findings from this study should help open the door for further studies on these promising compounds and on the derivatization of current drugs with organometallic moieties as well as on future designs of new complexes as possible antimicrobial agents with novel mechanisms of action.

## Figures and Tables

**Table 1:** Organometallic complexes in vitro antiparasitic activity against different micro-organisms.

Compound	Tested Micro-Organism	IC_50_ Value (μM)	Reference
**1**	*Trypanosoma cruzi*	0.25 ± 0.08	[23]
	*T. bruceibrucei* (strain 427)	0.6 ± 0.1	[23]
**2**	*Mycobacterium tuberculosis* (*Mtb*)	2.8 ± 0.7	[25]
	*T. cruzi*, Dm28c strain	0.64 ± 0.03	[25]
**3**	*Mycobacterium tuberculosis* (*Mtb*)	1.6 ± 0.3	[25]
	*T. cruzi*, Dm28c strain	0.28 ± 0.01	[25]
**4a**	*T. cruzi*	7.58 ± 0.10	[31]
	*T. brucei*	0.90 ± 0.05	[31]
**4b**	*T. cruzi*	1.42 ± 0.02	[31]
	*T. brucei*	0.93 ± 0.03	[31]
**4c**	*T. cruzi*	3.6 ± 0.05	[31]
	*T. brucei*	0.98 ± 0.06	[31]
**4d**	*T. cruzi*	29.4 ± 2.03	[31]
	*T. brucei*	1.56 ± 0.04	[31]
**5a**	*T. cruzi*	3.11 ± 0.05	[31]
	*T. brucei*	0.77 ± 0.02	[31]
**5b**	*T. cruzi*	0.79 ± 0.06	[31]
	*T. brucei*	0.60 ± 0.03	[31]
**5c**	*T. cruzi*	0.76 ± 0.03	[31]
	*T. brucei*	0.52 ± 0.03	[31]
**5d**	*T. cruzi*	1.32 ± 0.21	[31]
	*T. brucei*	1.01 ± 0.04	[31]
**6**	*Leishmania major*	2.0 ± 0.3	[32]
	*T. brucei*	0.7 ± 0.1	[32]
**7**	*Leishmania major*	3.6 ± 2.0	[32]
	*T. brucei*	13.2 ± 1.2	[32]
**8**	*Leishmania major*	1.8 ± 0.2	[32]
	*T. brucei*	0.4 ± 0.4	[32]
**9**	*Leishmania major*	4.7 ± 0.1	[32]
	*T. brucei*	2.1 ± 1.9	[32]
**10**	*Leishmania major*	2.2 ± 0.2	[32]
	*T. brucei*	0.5 ± 0.4	[32]
**KTZ**	*Leishmania major*	66.0 ± 3.9	[32]
	*T. brucei*	20.5 ± 0.4	[32]
**MCZ**	*Leishmania major*	42.6 ± 7.4	[32]
	*T. brucei*	17.5 ± 0.6	[32]
**CTZ**	*Leishmania major*	64.6 ± 2.1	[32]
	*T. brucei*	17.4 ± 0.6	[32]
**11–15**	*T. cruzi* trypomastigotes	0.3 ± 0.06	[33]
	*T. cruzi* Y strain	0.64 ± 0.1	[33]
**16**	*P. falciparum strains* NF54 (CQS)	1.93 ± 0.04	[35]
	*P. falciparum strains* Dd2 (CQR)	2.69 ± 0.22	[35]
**17**	*P. falciparum strains* NF54 (CQS)	1.81 ± 0.11	[35]
	*P. falciparum strains* Dd2 (CQR)	1.73 ± 0.16	[35]
**18**	*P. falciparum strains* NF54 (CQS)	1.76 ± 0.074	[35]
	*P. falciparum strains* Dd2 (CQR)	1.59 ± 0.053	[35]

**Table 2:** Antibacterial activity of the compounds against Gram-positive bacterial strains (the maximum concentration tested was 512 µg mL^−1^).

Compound	MIC					
	*B. subtilis*		*S. aureus* DSM 20231		*S. aureus* ATCC43300 (MRSA)	
	µg mL^−1^	µM	µg mL^−1^	µM	µg mL^−1^	µM
**19**	2	1.4	2	1.4	2	1.4
**20**	32	21	4	2.7	6	4
**21**	4	3	2	1.5	2	1.5
**22**	4	2.9	4	2.9	2	1.5
**23**	4	3.3	2	1.6	2	1.6

**Table 3:** MIC values of the mpo compounds against *M. tuberculosis* H37Rv as compared to MIC values of first- and second-line anti-TB drugs.

Compound	MIC Value against *M. tuberculosis* H37Rv (µM)	Reference
**29**	1.06	[17]
**30**	3.29	[17]
**31**	1.53	[17]
Isoniazid	0.18	[41]
Rifampicin	0.49	[41]
Pyrazinamide	48.74–406.14 ^a^	[41]
Ethambutol	2.45	[41]

^a^ At pH 5.5.

**Table 4:** Data on the antibacterial activity of **32**–**34**, and free dithiocarbamic acids (**LH**) (100 μg/mL).

Compound for Treatment	Inhibition Zone in mm			
	*B. cereus*	*E. coli*	*S. pneumoniae*	*V. cholerae*
**L^1^H**	4	3	2	3
**L^1^H**	7	4	3	3
**L^1^H**	5	4	3	6
**32**	5	7	5	6
**33**	14	13	13	15
**34**	9	8	7	9
Chloramphenicol	30	19	20	28
DMF	0	0	0	0

## Data Availability

Data is openly available and no new data were created for this study.
